# Development and evaluation of a societal core robotic surgery accreditation curriculum for the UK

**DOI:** 10.1007/s11701-024-02062-x

**Published:** 2024-08-06

**Authors:** Matthew W. E. Boal, Asma Afzal, Jack Gorard, Aishwarya Shah, Freweini Tesfai, Walaa Ghamrawi, Matthew Tutton, Jawad Ahmad, Chelliah Selvasekar, Jim Khan, Nader K. Francis

**Affiliations:** 1 The Griffin Institute, Northwick Park & St Marks’ Hospital, London, UK; 2 Wellcome/ESPRC Centre for Interventional Surgical Sciences (WEISS), University College London (UCL), London, UK; 3 The Association of Laparoscopic Surgeons of Great Britain and Ireland, London, UK; 4 The Royal Devon and Exeter NHS Foundation Trust, Exeter, UK; 5 University College London, London, UK; 6 East Suffolk and North Essex NHS Foundation Trust, Ipswich, UK; 7 University Hospitals Coventry and Warwickshire NHS Trust, Coventry, UK; 8 The Christie NHS Foundation Trust, Manchester, UK; 9 Portsmouth Hospitals University NHS Trust, Portsmouth, UK; 10 Division of Surgery and Interventional Science, Research Department of Targeted Intervention, UCL, London, UK; 11 Yeovil District Hospital, Somerset Foundation NHS Trust, Yeovil, UK

**Keywords:** Robotics, Training, Curriculum, Evaluation, Proficiency-based progression, Surgery, Development

## Abstract

Standardised proficiency-based progression is the cornerstone of safe robotic skills acquisition, however, is currently lacking within surgical training curricula. Expert consensuses have defined a modular pathway to accredit surgeons. This study aimed to address the lack of a formal, pre-clinical core robotic skills, proficiency-based accreditation curriculum in the UK. Novice robotic participants underwent a four-day pre-clinical core robotic skills curriculum incorporating multimodal assessment. Modifiable-Global Evaluative Assessment of Robotic Skills (M-GEARS), VR-automated performance metrics (APMs) and Objective Clinical Human Reliability Analysis (OCHRA) error methodology assessed performance at the beginning and end of training. Messick’s validity concept and a curriculum evaluation model were utilised. Feedback was collated. Proficiency-based progression, benchmarking, tool validity and reliability was assessed through comparative and correlational statistical methods. Forty-seven participants were recruited. Objective assessment of VR and dry models across M-GEARS, APMs and OCHRA demonstrated significant improvements in technical skill (*p* < 0.001). Concurrent validity between assessment tools demonstrated strong correlation in dry and VR tasks (*r* = 0.64–0.92, *p* < 0.001). OCHRA Inter-rater reliability was excellent (*r* = 0.93, *p* < 0.001 and 81% matched error events). A benchmark was established with M-GEARS and for the curriculum at 80%. Thirty (63.82%) participants passed. Feedback was 5/5 stars on average, with 100% recommendation. Curriculum evaluation fulfilled all five domains of Messick’s validity. Core robotic surgical skills training can be objectively evaluated and benchmarked to provide accreditation in basic robotic skills. A strategy is necessary to enrol standardised curricula into national surgical training at an early stage to ensure patient safety.

**Supplementary Information:**

The online version contains supplementary material available at 10.1007/s11701-024-02062-x.

## Introduction

Robotic surgery has evident advantages of improved dexterity, visualisation, and ergonomics. There are now over twenty current and emerging robotic surgery platforms and Intuitive Surgical have reported a milestone of over 13 million procedures [1] since its introduction, reflecting the rapid uptake of this technology. International surgical bodies [2–12] have made efforts to develop robotic curricula, however, due to rapid global expansion of platforms there is an increase in demand for standardisation of proficiency-based training in robotic surgery for the safe transfer of robotic skills to the operating room.

There have been multiple consensuses from robotic experts resulting in a well-defined framework for societies and specialties to follow [2, 3, 6, 10, 11, 13–17], however, implementation of curricula is yet to penetrate training ubiquitously. There are efforts, however, within multiple specialties [7, 18] to incorporate standardised, proficiency-based curriculum including the Fundamentals of Robotic Surgery by the Society of American Gastrointestinal and Endoscopic Surgeons (SAGES) [4] and the European Society of Coloproctology (ESCP) “ColoRobotica” pathway [19]. There is a plethora of research describing the development of virtual reality (VR) and specialty-specific curricula often only within expert centres [8, 18, 20].

**Fig. 1:**
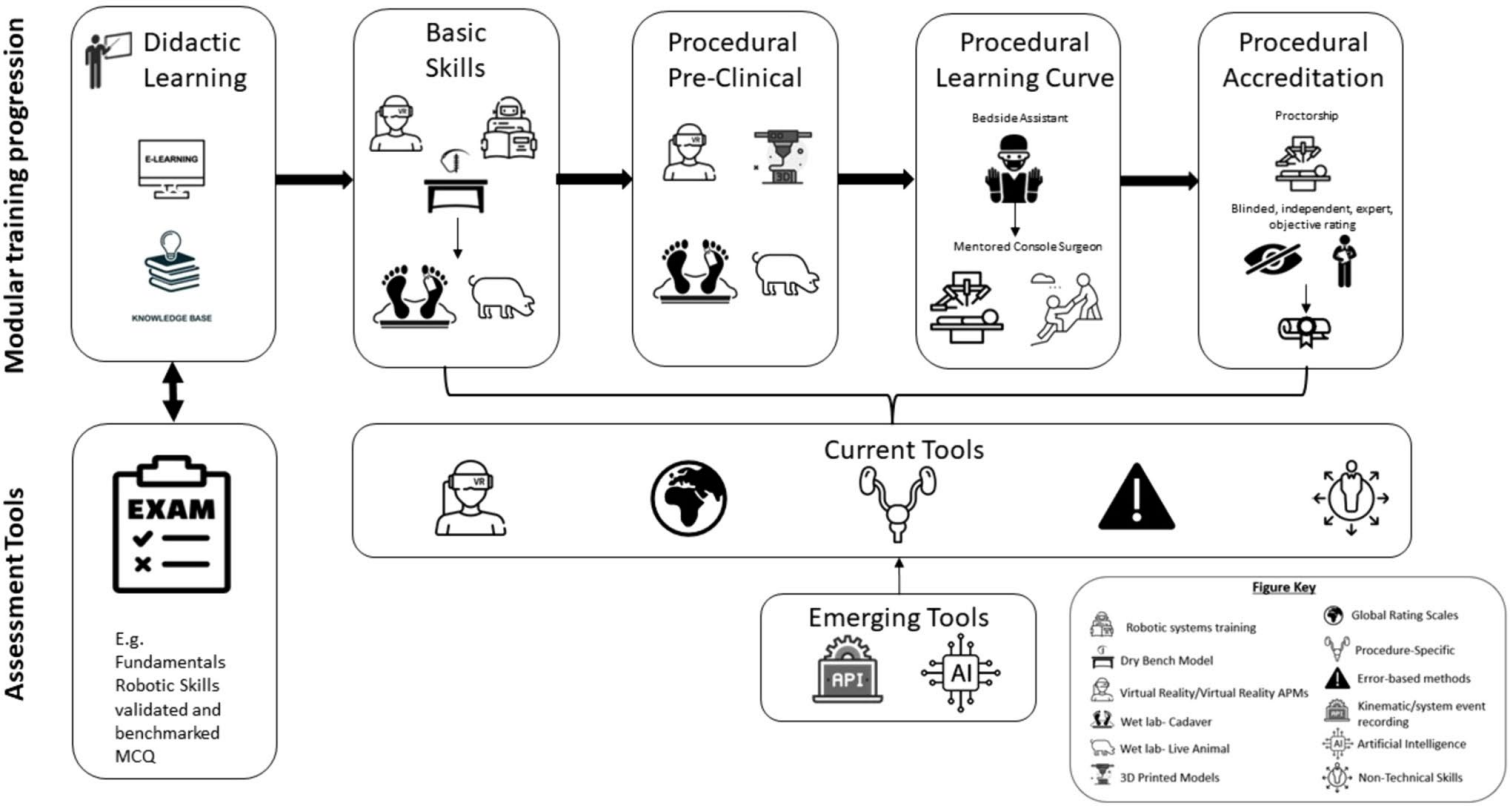
A standardised modular framework for a robotic surgery training curriculum

The generic curriculum framework (Fig. 1) consists of modular training incorporating knowledge-based didactic learning, device training, simulation including VR, dry and wet laboratory basic technical and non-technical skills, and finally procedural competency with mentor- and proctorships.

At the heart of this lies objective formative and summative assessment ensuring proficiency-based progression (PBP) and accreditation (PBA). There is a scarcity of comprehensive curricula [19, 21] that encompasses VR, dry and wet lab, with competency-based assessment that can benchmark core robotic surgical skills prior to procedural-based training. In addition, full evaluation of curricula is rare within surgical training [8, 18, 20], although not absent [3–5, 22].

In recognition of the educational need for a structured, comprehensively evaluated robotic training programme, the Association of Laparoscopic Surgeons of Great Britain and Ireland (ALSGBI), a minimal access surgical (MAS) society, has developed an accreditation-based programme for core robotic skills. This study aimed to report on the development and evaluation of a pre-procedural core robotic surgical skills curriculum.

## Methods

### Curriculum development and training

A steering group was assembled from robotic and educational experts from the ALSGBI robotic subcommittee. The literature was reviewed of previously defined curricula structures [2, 7, 8, 10, 11, 14–16, 18, 23, 24] and drafted the framework of the programme including task selection, assessment tools and evaluation.

Surgeon participants were recruited through the society’s course applications, screened by the robotic subcommittee, and non-surgeon doctors were selected from a university postgraduate masters’ degree in surgical sciences. They were invited to 4-day programmes between January 2022 and July 2023 at a pre-clinical surgical training hub. Helsinki principles were followed, collecting informed consent, alongside demography and surgical experience data prior to undertaking the curriculum. Training was provided on three, fourth generation da Vinci robots, with three VR simulator stations (da Vinci SimNow and da Vinci Surgical Skills Simulator). Device and systems-based knowledge was provided through a pre-course educational booklet and a phone app with a validated curriculum [4, 25]. Practical training included technical skills drills on VR, dry and wet ex-vivo porcine small bowel and avian vessel dissection [26] simulation, docking, undocking, troubleshooting, non-technical skills and emergency scenarios. All assessments were performed on the Xi and da Vinci SimNow.

### Tasks

**Fig. 2:**
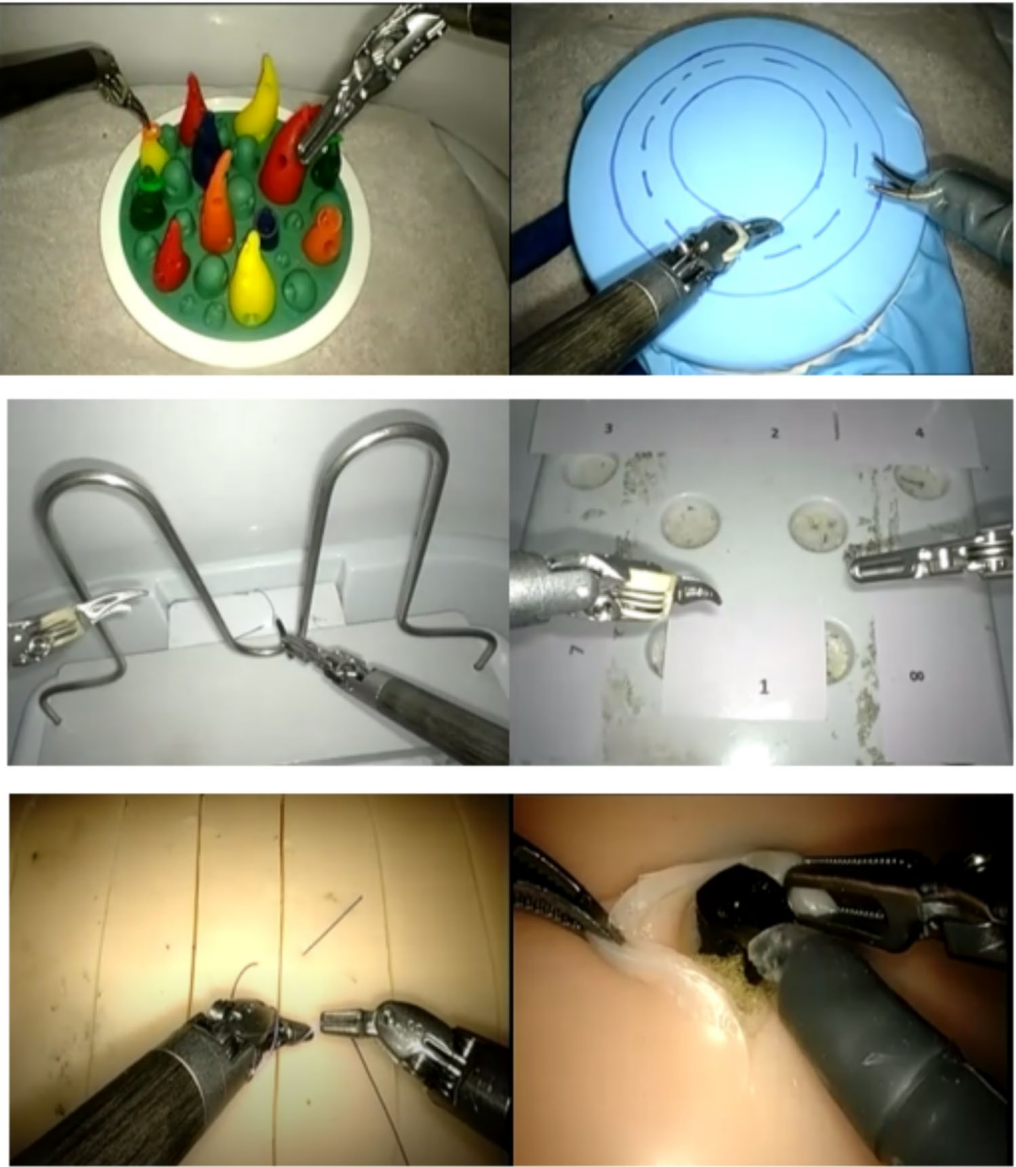
Dry tasks (top left to bottom right): Sea spikes, glove cutting, ring rollercoaster, camera targeting relay, interrupted suture and cyst excision model

The dry tasks assessed were sea spikes, ring rollercoaster, glove cutting, interrupted suture with an additional 5th task of camera target relay on the most recent course (Fig. 2) and were benchmarked in this study. The VR tasks assessed in the curriculum were similar to the dry models.

### Curriculum evaluation

Following Kirkpatrick’s model [27, 28] of curriculum evaluation, data were collated using a standardised questionnaire to assess participants’ immediate reaction through feedback (level 1). PBP was objectively assessed (level 2) by trained, expert raters in a formative session on day one at the beginning (attempt 1) and a summative exam at the end of the week on day 4 (attempt 2). The exam consisted of the dry lab tasks plus a silicone cyst model. In addition, a bespoke docking/undocking checklist with a non-technical skills assessment, Interpersonal and Cognitive Assessment for Robotic Surgery (ICARS) [29], was utilised and a pass/fail assigned (Table 1). Finally, a 20-question multiple-choice (MCQ) exam was sat by the participants. Formal evaluation of Kirkpatrick’s level 3 and 4 lay outside the scope of this project as it is a pre-clinical curriculum. However, a survey was sent to surgeon participants 6 months after completing the course in an attempt to evaluate the impact of the programme on several aspects of non-technical skills, systems knowledge, docking, assisting, safe console operating, troubleshooting and emergency scenarios.

**Table 1:** A bespoke docking and undocking checklist (pass/fail)

Candidate name:		
Date and AM/PM:		
Docking step	0/1	General Comments stating the step if appropriate
(1) Sterile field including draping, following protocol		
(2) Clear staff brief, assign roles including for emergency undocking, confirm reverse communication, points of reference and target anatomy		
(3) Appropriate OR set up. Candidate aware of each component, function, and placement. Vision cart, patient cart and surgeons' console		
(4) Patient cart appropriately positioned, depending on robot generation		
(5) Safe insertion of endoscope port 10-20 cm from target anatomy		
(6) Pneumoperitoneum		
(7) Identify surgical workspace, ensure target anatomy is central		
(8) Safe (direct vision) and correct insertion of subsequent ports i.e. correct depth, 4–8 cm between, parallel line, perpendicular to target anatomy, ≥ 2 cm from bony prominences		
(9) Ensure patient bed is positioned/tilted if needed now before attaching ports (unless Xi with paired operating bed)		
(10) Moving patient cart to bedside, correct approach side and green laser alignment with endoscope/target anatomy*		
*If Xi, press and hold deploy for docking before approach, to automatically move boom to correct position before moving to patient bedside, guided by green laser cross lining up with endoscope port		
(11) Safe, appropriate, clear communication whilst docking with minimum two people, including establishing room references for directed movement		
(12) Dock first to endoscope port and insert endoscope*		
*If Xi, aim at target anatomy and use target anatomy button to automatically move boom, ensuring to stabilise endoscope port with one hand for safety		
(13) Dock remaining arms to ports		
(14) Recheck ports remain set to correct depth and aiming towards target anatomy, cheque ports for skin pinching and “bounce” if necessary		
(15) Final system walk around, checking for outside clashing, minimum fist distance between arms		
(16) Safe instrument insertion under direct vision (assistant or surgeon controlling endoscope). Ensure reverse communication between surgeon at console and assistant inserting		

Undocking step (changing)	Score	General Comments
(1) Surgeon confirms safe removal of instruments, making sure instruments straight and not holding tissue before removal exiting, assistant and surgeon must visualise this, again reverse communication		
(2) Undock arms from port cannulas. Remove up and away from patient		
(5) Move patient cart away from beside correctly, ensuring communicating this with the team, and checking for obstacles including clashing with the patient		
(4) Patient cart stowed correctly, automatic feature if Xi		
Overall score (including questions score of 5)		
Pass or fail? (1 = pass, 0 = fail) NB this has not been benchmarked, more your (the assessors) general feeling		

Score key:

0 = Not completed 1 = Completed

Objective assessment tools implemented were Modifiable-Global Evaluative Assessment of Robotic Skills (M-GEARS) (Appendix- Supplementary Figure_1), modified by our group from the original GEARS [30] to address missing domains key to generic robotic skills [31]. The modification of the tool was underpinned by reviewing the literature and identifying other objective assessment tools that evaluated these missing items, including Assessment of Robotic Console Skills (ARCS) [32] and Objective Structured Assessment of Technical Skills (OSATS). Virtual reality automated performance metric (APM) scores from the da Vinci SimNow simulator were recorded. To objectively assess errors within basic robotic technical skills, a bespoke Objective Clinical Human Reliability Analysis (OCHRA) tool was developed from a previous evaluated clinical OCHRA methodology [33–37] and applied through blinded, retrospective video analysis [38]. Concurrent validity of OCHRA with M-GEARS and VR scores had already been established in previous work during this curriculum, as well as excellent inter-rater reliability [38].

To further evaluate the curriculum, Messick’s concept was utilised, viewing validity as a continuous process with five domains to be achieved for comprehensive evaluation [39, 40]:

1. Test content: Demonstrated through expert and guideline driven curriculum development.
2. Response process: Demonstrated through expert, trained assessors and blinded, independent video review.
3. Internal structure: Evaluated in reliability analyses and the use of inherently reliable automated metrics from the VR simulator.
4. Relationship to other variables: Evaluated with traditional concepts of concurrent/construct validity and proficiency-based progression using objective assessment tools.
5. Consequence: Demonstrated through the evaluation of a benchmark based on the results and previous literature and through a survey.

### Statistical analysis

Participants’ progression was analysed using a paired t-test to compare the mean of M-GEARS, VR and OCHRA scores on attempt 1 and attempt 2. Due to the sample size number for the new fifth task, camera targeting, a Wilcoxon Signed Rank test was run. A Kruskal–Wallis ANOVA test compared consultants, senior surgical trainees and non-surgeon junior doctors mean scores to investigate construct validity. A pass mark of 80% was defined and consisted of a cumulative score from the MCQ (20%), attempt 2 of the dry tasks (40%) and the cyst excision model (40%) M-GEARS scores. Those who failed were offered a further training day and to retake the exam within six months. To benchmark M-GEARS, the total summative technical skills scores were compared in the pass and fail groups using an independent samples *t* test. All analysis was performed on IBM SPSS Statistics 28 directed and ratified by an independent university lecturer statistician.

## Results

Forty-seven participants, 27 (57%) males and 20 (43%) females, underwent the pre-procedural robotic skills curriculum, across seven courses. There was a mean age of 32 (range 23 to 62). 16 (34%) were classified as senior surgeons (registrars, senior fellows, consultants) and 31 (66%) junior doctors. All participants were considered novice robotic surgeons, with no prior experience, except one consultant who had minimal console experience (ten operations). The senior surgeons had extensive laparoscopic experience, all juniors had none or very little i.e. had only assisted and one junior had attended a robotic course. Seven senior surgeons had attended basic robotic skills courses in the past, and six had assisted in robotic surgery (mean 16.67 cases (range 3–50).

### Participant reaction

Participant feedback (Supplementary Figure_2) was collected from all 47 participants, with overall rating averages at 5 out of 5 and 100% recommended the programme. Improvements pertained to further procedure-specific module availability and logistical issues.

### Objective assessment and proficiency-based progression

**Fig. 3:**
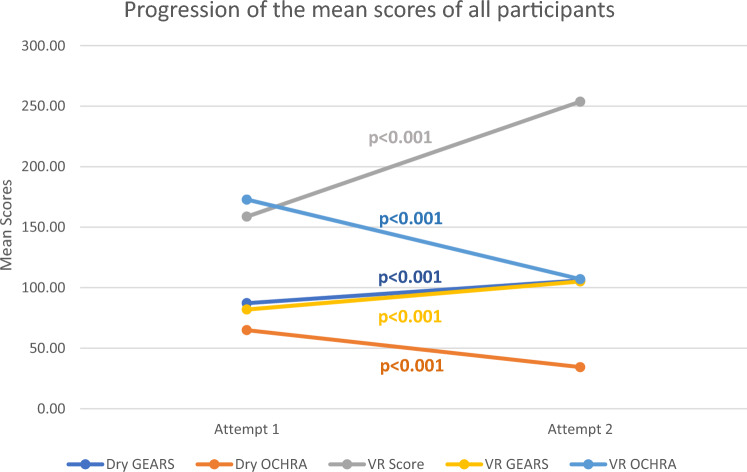
Line graph demonstrating the mean sum scores of all participants’ progression in each assessment domain from attempt 1 to attempt 2

The mean dry assessment scores below demonstrate improvements for all participants from attempt 1 to 2 (Fig. 3 and Table 2). Participants’ dry M-GEARS scores (*n* = 47) for all four tasks demonstrated a mean difference of 18.72 (SD 20.38, 95% CI 12.74–24.71, *p* < 0.001) and the newly introduced fifth dry lab task (*n* = 9), camera targeting relay, showed a significant improvement (*p* < 0.017). Dry tasks’ OCHRA assessments demonstrated a significant reduction in participants’ (*n* = 35) errors with a mean difference of –30.57 (SD 31.36, 95% CI – 41.34 to – 19.80, *p* < 0.001). The mean scores of all participants VR assessments all significantly improved from attempt 1 to 2 (Fig. 3 and Table 2). VR-automated scores (*n* = 35) demonstrated a mean difference of 98.25 (SD 68.75, 95% CI 74.64–121.87, *p* < 0.001). Virtual reality M-GEARS scores (*n* = 35) mean difference was 24.10 (SD 11.45, 95% CI 20.17–28.03, *p* < 0.001) and VR OCHRA error scores (*n* = 34) improved with a mean difference of – 69.67 (SD 60.83, 95% CI – 90.89 to – 48.45, *p* < 0.001).

**Table 2:** Paired *t* tests comparing the means of attempt 1 and 2 for each assessment category

	n	Paired Differences					*t*	df	Significance
		Mean	SD	SE	95% Confidence Interval of the Difference				*p* value
					Lower	Upper			
Sum Dry M-GEARS attempt 1 Sum Dry M-GEARS attempt 2	47	18.723	20.384	2.973	24.708	12.738	6.297	46	< 0.001
Sum Dry camera targeting M-GEARS attempt 1 Sum Dry camera targeting M-GEARS attempt 2	9	6.889	5.442	1.814	11.072	2.706	3.798	8	0.005
Sum Dry OCHRA attempt 1 Sum Dry OCHRA attempt 2	35	– 30.571	31.355	5.300	-19.801	– 41.342	– 5.768	34	< 0.001
Sum VR-automated scores Attempt 1 Sum VR-automated scores attempt 2	35	98.257	68.751	11.621	121.874	74.640	8.455	34	< 0.001
Sum VR GEARS attempt 1 Sum VR GEARS attempt 2	35	24.100	11.454	1.936	28.034	20.166	12.448	34	< 0.001
Sum VR OCHRA attempt 1 Sum VR OCHRA attempt 2	34	– 69.676	60.825	10.431	– 48.453	– 90.899	– 6.679	33	< 0.001

**Fig. 4:**
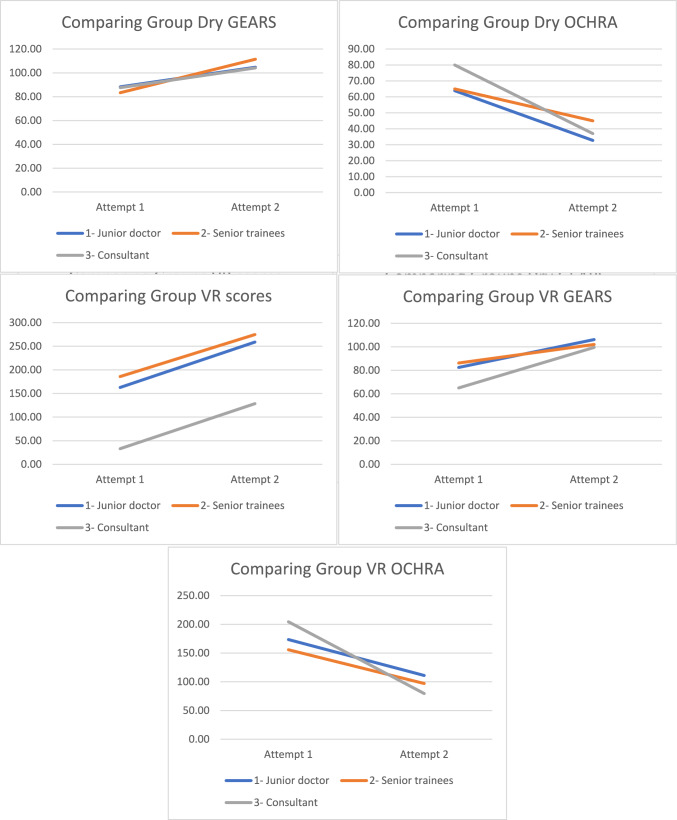
Comparing three participant groups across assessment tools- junior doctors (1), senior trainees/fellows (2) and consultants (3)

Construct validity was investigated comparing groups, demonstrating no significant differences in performance across domains at both attempts (Fig. 4 and Table 3).

**Table 3:** Independent samples Kruskal–Wallis test between junior doctors (non-surgeons), senior surgical trainees and consultant surgeons

Assessment domain and task	*n*	Test statistic	df	*p* value
Dry M-GEARS attempt 1	47	0.418	2	0.811
Dry OCHRA attempt 1	35	0.878	2	0.645
Dry M-GEARS attempt 2	47	1.183	2	0.553
Dry OCHRA attempt 2	35	1.865	2	0.394
VR-automated score attempt 1	36	3.568	2	0.168
VR M-GEARS attempt 1	35	5.286	2	0.071
VR OCHRA attempt 1	36	0.753	2	0.686
VR-automated score attempt 2	35	5.049	2	0.08
VR M-GEARS attempt 2	35	1.257	2	0.533
VR OCHRA attempt 2	34	1.197	2	0.55

A moderate to strong correlation (*n* = 47, *r* = 0.51, 95% 0.26–0.70, *p* value < 0.001) was demonstrated between the sum scores of attempt two dry lab tasks and the summative cyst removal (Supplementary Figure_3).

Six of the surgeon participants (37.5%) responded to the evaluation survey, reporting significant improvements (*p* = 0.026 to 0.042) across the domains questioned for skill retention and long-term impact. Thirty of the total 47 participants (63.82%), with 13 of 16 surgeons, achieved the pass mark of 80% or over. Mean total M-GEARS scores for the summative dry tasks second attempt and the cyst model were compared between pass and fail groups. The means were 86.05% and 70% for pass and fail participants respectively (mean difference 16.05, SE 2.06, 95% CI 11.90–20.22, *p* < 0.001).

## Discussion

This study has developed and objectively evaluated a comprehensive, society-led, pre-clinical core robotic surgery curriculum which provides a blueprint for further cross specialty and alternate robotic platforms training, in addition to existing curricula [4, 19]. It incorporates multiple modules of the robotic training pathway, implementing validated formative objective skill and error assessment, with full evaluation according to Messick’s domains (Table 4) and short-term outcomes of Kirkpatrick’s model [27, 28, 41].

**Table 4:** Messick’s validity concept description and curriculum evidence

Source of validity	Description	Curriculum evidence
Test Content i.e. face and content validity	The test’s content and the construct it is intended to measure	Developed from established international expert consensuses
Response process	Analysis of raters and how well they respond to the test with steps taken to improve the validity	Raters were orientated to the M-GEARS tool and trained with an expert in its use with initial dual rating and scoring consensus Virtual reality automated performance metrics (VR APMs) eliminate rater bias
Internal structure	Degree to which domains and aspects of the tool fit the underlying construct	GEARS has been shown to have high to excellent reliability in the majority of studies[42] VR APMs are inherently reliable OCHRA demonstrated excellent IRR and high matched error consensus agreement
Relationship to other variables	Evaluating scores’ associations/correlations, whether they are positive or negative, strong or weak, and with other variables including discriminative ability	Proficiency-based progression was demonstrated in all objective assessment tools across dry and VR tasks Concurrent validity was established with strong correlations between objective assessment tools Construct was investigated and showed no significant difference between groups, which is arguably unsurprising given that all participants were robotic novices Predictive validity is lacking in this young curriculum, however, it is not unreasonable to assume that participants who passed would be safe to go on to supervised, live operating
Consequence	Impact of the assessment	Pass/fail the whole curriculum was established with expert consensus and benchmarking of the M-GEARS assessment tool An evaluation survey demonstrated weak evidence of skill retention and long-term impact

There are a number of published robotic curricula, most are VR-based, with few reports on dry lab programmes that are proficiency-based and validated [18]. There is, however, as the uptake of robotic surgery and training increases, more evidence of competency-based curriculum evaluation emerging [21]. Virtual reality is a useful adjunct in the understanding of robotic functions and the safe acquisition of basic generic skills but lacks those learnt from dry and wet lab including force sensitivity, visual haptic feedback, instrument clashing and non-technical skills. Therefore, this curriculum was designed with the aim to address all those needs along with objective assessment of skills and errors in each generic robotic skill domains, that is, depth perception, bimanual dexterity with multiple instruments, force sensitivity, autonomy, master manipulator workspace, camera control and basic energy skills. The curriculum design was based on previously established and expert defined parameters for robotic basic skills training [2, 4, 7, 15, 16, 25, 43], lending it face and content validity (Messick’s test content). The components included to evaluate participants satisfied the recent consensus requirements of a pre-procedural core robotic surgery curriculum [16]. For full evaluation of robotic surgery curricula, careful methodology and reliable objective assessment of surgical technical and non-technical performance is essential. Within the United Kingdom (UK) the British Association of Urological Surgeons (BAUS) have developed comprehensive programmes for trainees to reach competency [7], however, despite being early adopters of robotic surgery, fellowships lack the implementation of validated, objective assessment tools in the formative and summative setting.

Considering Messick’s validity domains of response process and internal structure, APM scores from the da Vinci SimNow can be considered inherently objective, and reliable. Although further validation efforts are required [44, 45] similar to the evaluation of older VR simulators [42]. OCHRA inter-rater reliability was established as excellent. GEARS reliability has not been formally evaluated here; however, trainers were senior surgeons with experience of the tool, receiving orientation with initial consensus discussions and dual rating at the start of use, to align scoring. Although human manual rating is inherently subjective and with reports of low reliability, many studies have concluded GEARS to have excellent reliability [42].

Error-based methods of surgical skill and performance within robotics lack granularity and have not been fully evaluated. OCHRA, however, has been validated within laparoscopy [33, 34, 37] and therefore intuitively, it should be transferable to robotic surgery. This curriculum has demonstrated for the first time OCHRA’s feasibility of use in robotic technical skills assessment [38], objectively assessing the participants’ progression, fulfilling Messick’s “relationship to other variables”. Within this domain, the proficiency-based progression of participants has been objectively assessed with three tools from different technical skills assessment domains: manual, VR-automated performance metrics and error methods. Our additional research in the application of OCHRA in the pre-clinical setting [38] demonstrated its potential as a formative and summative tool, demonstrating concurrent validity, excellent inter-rater reliability and highlighting technical errors. The commonest was “inappropriate/poor use of endowristed instrument”, and their consequences, for example “delay in progress of procedure” or “risk of injury/minor collision”. This granular feedback could provide insight to the training needs of surgeons.

To accredit surgeons in basic robotic knowledge and skills a pass/fail benchmark is recommended to allow safe progression to console training. Multiple studies have previously suggested benchmarks for manual and VR tool scores usually between 80 and 90%[42], and the Fundamentals Robotic Surgery (FRS) VR curriculum benchmarked their multiple-choice exam through experts’ mean scores at 39 out of 44 (88.64%). This is available for free on the Institute for Surgical Excellence website or phone app, with the included online learning material [25]. As the curriculum developed we incorporated this into the programme alongside a benchmarked VR competency of 90% in 10 tasks assessing the key technical skill domains highlighted previously. A survey was sent to all surgeon participants to evaluate skill retention and long-term impact as part of Messick’s consequence domain and level 3 and 4 of Kirkpatrick’s model.

A secondary aim of this study was to validate our modification of GEARS, which has been achieved demonstrating proficiency-based progression, concurrent validity and benchmarking across all domains of assessment and additional items within the tool i.e. basic energy skills, flow of operation and 3rd arm use. In previous studies, GEARS has not been thoroughly or transparently benchmarked with scores between 80 and 100% and 2.9 out of 5 in each domain [42]. Our curriculum suggests, from the mean summative scores of the pass candidates, that  86% of the total Modifiable-GEARS score should be the pass mark. For example, if the tool is out of 35, the pass mark should be 30. Some domains are often not scored, that is, autonomy in a blinded review, or energy skills in simple dry tasks, allowing the M-GEARS tool to be flexible. This benchmark is not dissimilar from other studies or scoring tools such as Objective Structured Assessment of Technical Skills (OSATS), novices scoring 3.5 and experts 4.5 out of 5, Global Operative Assessment of Laparoscopic Skills (GOALS) at 80% and Robotic-OSATS (R-OSATS) at 70% [42]. It was considered reasonable and consistent, therefore, to implement a pass mark for GEARS at 80% in dry simulation tasks.

### Limitations

Although this study has achieved its goals in terms of pre-clinical core robotic curriculum evaluation, it requires long-term studies to establish construct and predictive validity. In the future iterations we would recommend expanding the curriculum to other specialties such as thoracics and gynaecology where the uptake robotic surgery is increasing. This would be encompassed within Kirkpatrick’s levels 3 and 4 evaluation which address participant behaviour change and clinical impact/skill retention. An interesting finding within this study was that there was no significant difference between the levels of seniority and performance. This is not completely surprising as a surgical robot is a new tool and entirely different to any other within the healthcare setting, therefore, all participants can be considered novices. This introduces the argument that surgical residents should start robotic training at an early stage rather than as a post-certification fellowship.

Additional limitations existed within the confines of what was logistically feasible, that is, blinded rating for formative and summative sessions. Lastly, changes to the curriculum resulted in reduced participant numbers for the VR domains of analysis, and due to recording issues there was some missing data for one candidate’s attempt 1 VR and OCHRA scores.

## Conclusion

Core robotic surgical skills training can be objectively evaluated and benchmarked to provide accreditation in basic robotic skills. A strategy is necessary to enrol curricula such as this into a standardised, national surgical training at an early stage to ensure patient safety. This would include facilitating the expansion of other training hubs to enable nationwide standardised training in efforts to ensure patient safety.

## Supplementary Information

Below is the link to the electronic supplementary material.

Supplementary file1 (DOCX 619 KB)

## Data Availability

No datasets were generated or analysed during the current study. Data can be reproduced on reasonable request.
