# Supplementary material for: Development and evaluation of a societal core robotic surgery accreditation curriculum for the UK
Source: J Robot Surg. 2024 Aug 6;18(1):305. doi: 10.1007/s11701-024-02062-x (PMC11303427; doi:10.1007/s11701-024-02062-x)
Supplement: Supplementary file 1 — Supplementary file1 (DOCX 619 KB) [file 11701_2024_2062_MOESM1_ESM.docx]

**Development and Evaluation of a Societal Core Robotic Surgery Accreditation Curriculum for the UK**

**Authors:** Matthew WE Boal (MBChB)^1,2,3^, Asma Afzal (MD)^3,4^, Jack Gorard (MBChB)^5^, Aishwarya Shah (iBSc)^5^, Freweini Tesfai (MD/MSc)^1,2,3^, Walaa Ghamrawi (MD)^1,2^, Matthew Tutton (FRCS)^3,6^, Jawad Ahmad (FRCS)^3,7^, Chelliah Selvasekar (PhD, FRCS)^3,8^, Jim Khan (PhD, FRCS)^3,9^, Nader K Francis (PhD, FRCS)^1,3,10,11^

**Affiliations:**

1. The Griffin Institute, Northwick Park & St Marks’ Hospital, London, UK
2. Wellcome/ESPRC Centre for Interventional Surgical Sciences (WEISS), University College London (UCL), UK
3. The Association of Laparoscopic Surgeons of Great Britain and Ireland, UK
4. The Royal Devon and Exeter NHS Foundation Trust
5. University College London, UK
6. East Suffolk and North Essex NHS Foundation Trust, UK
7. University Hospitals Coventry and Warwickshire NHS Trust, UK
8. The Christie NHS Foundation Trust, UK
9. Portsmouth Hospitals University NHS Trust, UK
10. Division of Surgery and Interventional Science, Research Department of Targeted Intervention, UCL, UK
11. Yeovil District Hospital, Somerset Foundation NHS Trust, UK

**Conflict of interest and Source of Funding:**

No funding sources

Matt Boal’s PhD is funded by Digital Surgery Ltd, Medtronic

**Category:** Original article

**Word count**: 2741

**Author correspondence:** Professor Nader Francis, The Griffin Institute, Northwick Park and St Marks Hospital, London, UK. [N.francis@griffininstitute.org.uk](mailto:N.francis@griffininstitute.org.uk). 02039580500.

**Keywords:** Robotic, Surgery, Curriculum, Development, Evaluation, Proficiency

**Acknowledgements:**

Thank you to Digital Surgery Inc, a Medtronic Company, for funding Matthew Boal’s PhD.

Thank you to all participants of the curriculum.

Thank you to Dr C. Suarez-Rivera, UCL Associate Lecturer, for providing statistical consulting support.

**Data access:** Data can be reproduced on reasonable request

**Supplementary Figures:**

Supplementary Figure 1: Modifiable-GEARS assessment tool

Supplementary Figure_2: Example feedback capturing participants’ reaction

Supplementary Figure_3: Scatter plot demonstrating the correlation between attempt 2 dry task sum M-GEARS scores and the summative cyst removal final score


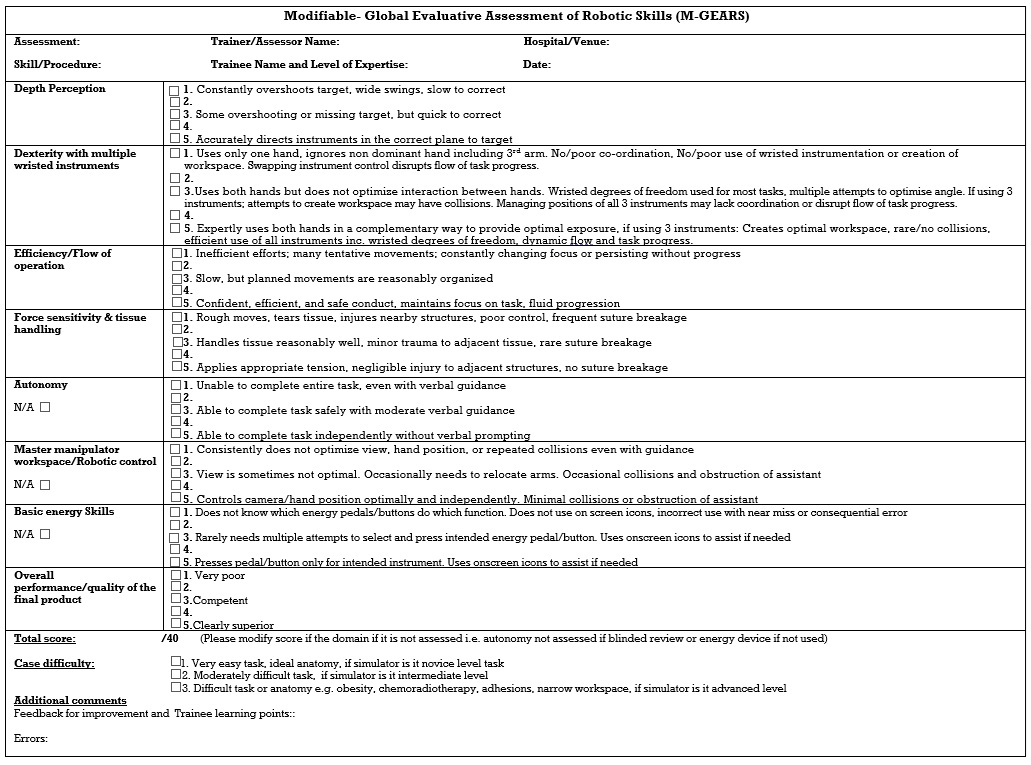
Supplementary Figure 1: Modifiable-Global Evaluative Assessment of Robotic Skills (M-GEARS) assessment tool

Supplementary Figure_2: Example feedback capturing participants’ reaction


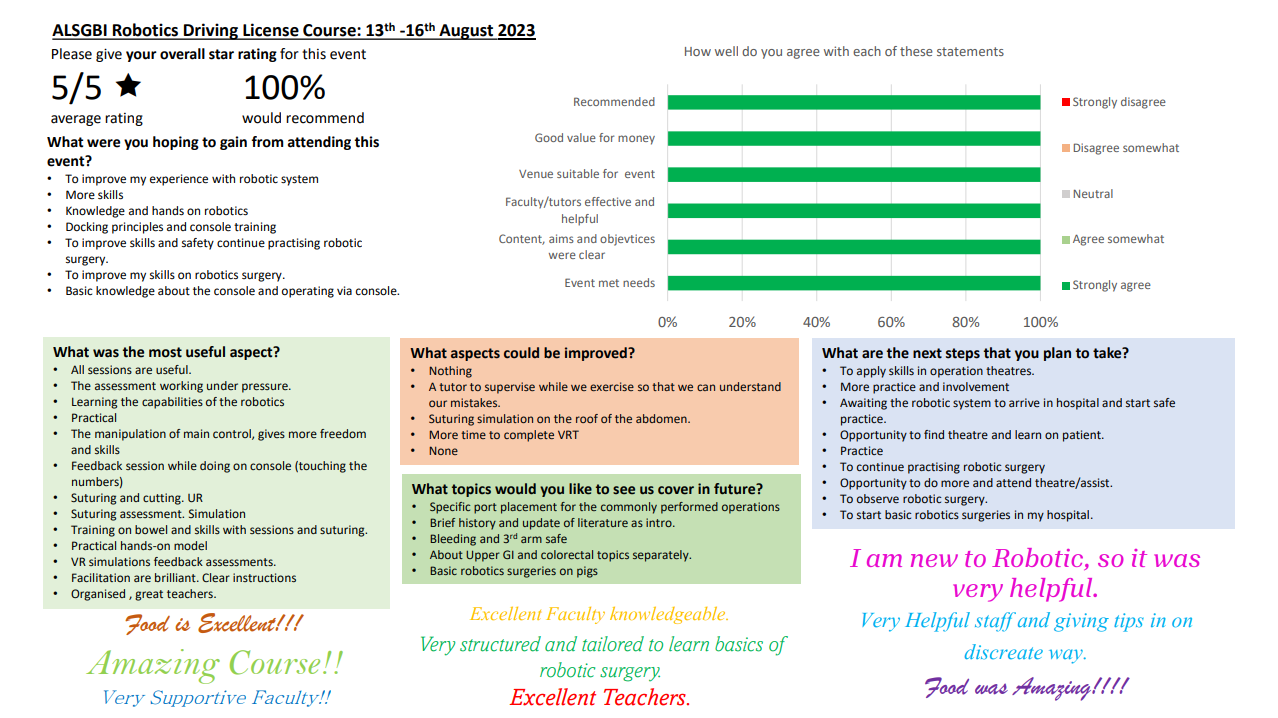


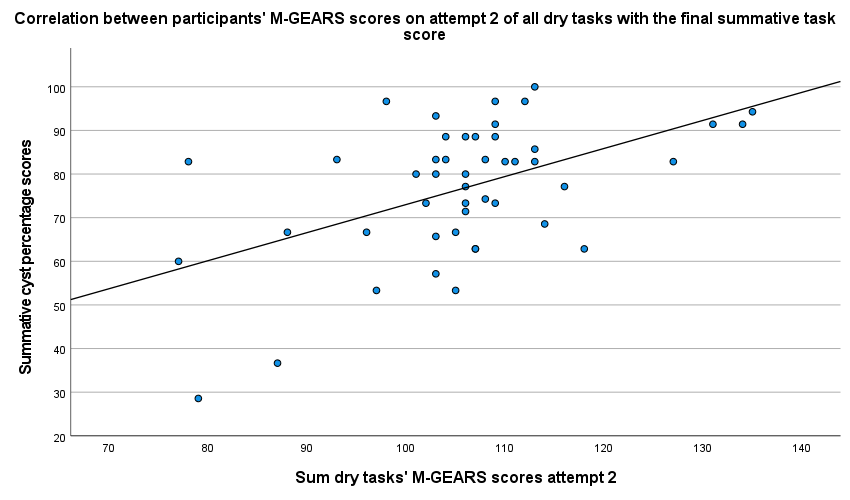
Supplementary Figure_3: Scatter plot demonstrating the correlation between attempt 2 dry task sum M-GEARS scores and the summative cyst removal final scores

Top of Form
